# Biocatalytic Buoyancy-Driven Nanobots for Autonomous Cell Recognition and Enrichment

**DOI:** 10.1007/s40820-023-01207-1

**Published:** 2023-10-24

**Authors:** Ziyi Guo, Chenchen Zhuang, Yihang Song, Joel Yong, Yi Li, Zhong Guo, Biao Kong, John M. Whitelock, Joseph Wang, Kang Liang

**Affiliations:** 1 School of Chemical Engineering, Australian Centre for NanoMedicine, The University of New South Wales, Sydney, NSW 2052 Australia; 2 Medical College, Northwest Minzu University, Lanzhou, 730000 People’s Republic of China; 3 General Intensive Care Unit, Second Affiliated Hospital of Zhejiang University School of Medicine, Hangzhou, People’s Republic of China; 4 School/Hospital of Stomatology, Lanzhou University, Lanzhou, 730000 People’s Republic of China; 5 Department of Chemistry, Shanghai Key Lab of Molecular Catalysis and Innovative Materials, Collaborative Innovation Center of Chemistry for Energy Materials, Fudan University, Shanghai, 200438 People’s Republic of China; 6 Graduate School of Biomedical Engineering, The University of New South Wales, Sydney, NSW 2052 Australia; 7 Department of Nanoengineering, University of California San Diego, La Jolla, CA 92093 USA

**Keywords:** Nanobots, Surface functionalization, Cell recognition, Cell separation, Metal–organic frameworks

## Abstract

Autonomously self-propelled nanoswimmers represent the next-generation nano-devices for bio- and environmental technology. However, current nanoswimmers generate limited energy output and can only move in short distances and duration, thus are struggling to be applied in practical challenges, such as living cell transportation. Here, we describe the construction of biodegradable metal–organic framework based nanobots with chemically driven buoyancy to achieve highly efficient, long-distance, directional vertical motion to “find-and-fetch” target cells. Nanobots surface-functionalized with antibodies against the cell surface marker carcinoembryonic antigen are exploited to impart the nanobots with specific cell targeting capacity to recognize and separate cancer cells. We demonstrate that the self-propelled motility of the nanobots can sufficiently transport the recognized cells autonomously, and the separated cells can be easily collected with a customized glass column, and finally regain their full metabolic potential after the separation. The utilization of nanobots with easy synthetic pathway shows considerable promise in cell recognition, separation, and enrichment.

**Supplementary Information:**

The online version contains supplementary material available at 10.1007/s40820-023-01207-1.

## Highlights

- A self-propelled, biocatalytic buoyancy-driven metal–organic framework (MOF) nanobot is developed.
- The anti-carcinoembryonic antigen antibody functionalized MOF nanobot demonstrates directional vertical motion to “find-and-fetch” cancer cells from mixed cell population.
- The captured cells can be recovered with full metabolic potential.

**Supplementary Information:**

The online version contains supplementary material available at 10.1007/s40820-023-01207-1.

## Graphical abstract

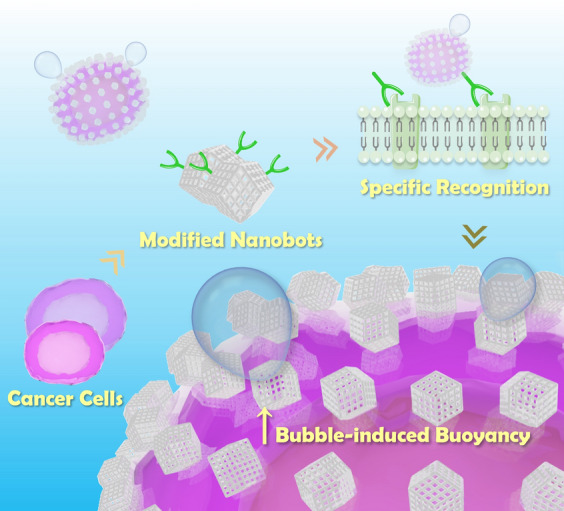

## Introduction

Cell separation is widely used in clinical therapy and many strands of biological research. The ability to sort cells from heterogeneous populations enables the further diagnosis of individual cell types [1, 2]. This technology underpins many key discoveries in cell biology and disease diagnostics and is further enabling research in areas as diverse as regenerative medicine, cancer therapy and HIV pathogenesis [3–7]. The development of micro/nano technology on cell manipulation and sorting were reviewed by Carlo and co-workers, which introduced several cell sorting mechanisms including inertial microfluidics, magnetic cell manipulation, electrical manipulation, optical manipulation, acoustic cell manipulation and immuno-microbubbles [8].

The well-studied microfluidic devices exhibited extensive applicability due to the flexibility in structure designs and integrating capacity with different mechanisms, for instance, microfiltration, deterministic lateral displacement, and pinched flow fractionation [9]. However, these devices usually depend on the physical properties of the cells [10, 11] (e.g., size [12], shape [13], deformability [14, 15], and dielectrophoretic responses [16, 17]), which resulted in inadequate separation purity and lacks sensitivity in specific cell phenotype isolation. To increase the recognizing specificity, immunomagnetic separation with antibody-functionalized particles exhibits great potential for specific cell recognition and collection [18–21]. However, the antibody-modified magnetic beads rely on external magnetic fields and additional manual manipulation, also the magnetic beads made it difficult to retrieve captured cells as the magnetic particles are permanently bound to the cells. On the other hand, the antibody-lipid microbubbles could be easily destroyed by quick sonication, but the clinical application was restricted by the storage and transporting stability. To mimic the biological immunological recognition process, automatic isolation systems with better specificity and stability are expected.

Due to the diverse morphology [22, 23], ease of motion manipulation [24, 25], and great biocompatibility [26, 27], chemically driven self-propelled artificial nanobots have shown considerable potential in various aspects ranging from drug delivery to water remediation [28–32, 55, 56]. Hitherto, only a few functionalized microrobots have been applied in specific isolation of biomacromolecules [33] and living entities [34–37]. For instance, bubble thrust propelled, tubular micromotors were decorated with antibodies [35] and poly(3-aminophenylboronic acid) [36] for cell recognition. In a later study, a magnetically driven helical microcarrier was developed for sperm cell transportation [37]. Although these micro-to-millimeter sized systems have achieved the primary goal of recognizing and carrying the targeted cells while moving, the ability for nanomachines to maneuver heavy objects collectively in longer distance has not been achieved. In addition, while existing works focused more on single cell manipulation, the autonomous collective transportation and enrichment of target cells from a mixed population is yet to be achieved. Success in this endeavor would allow the true phenotypical and metastatic potential of cells of interest to be isolated and studied, thus facilitating the development and utilization of nanobots in more realistic biomedical and clinical applications.

**Scheme 1:**
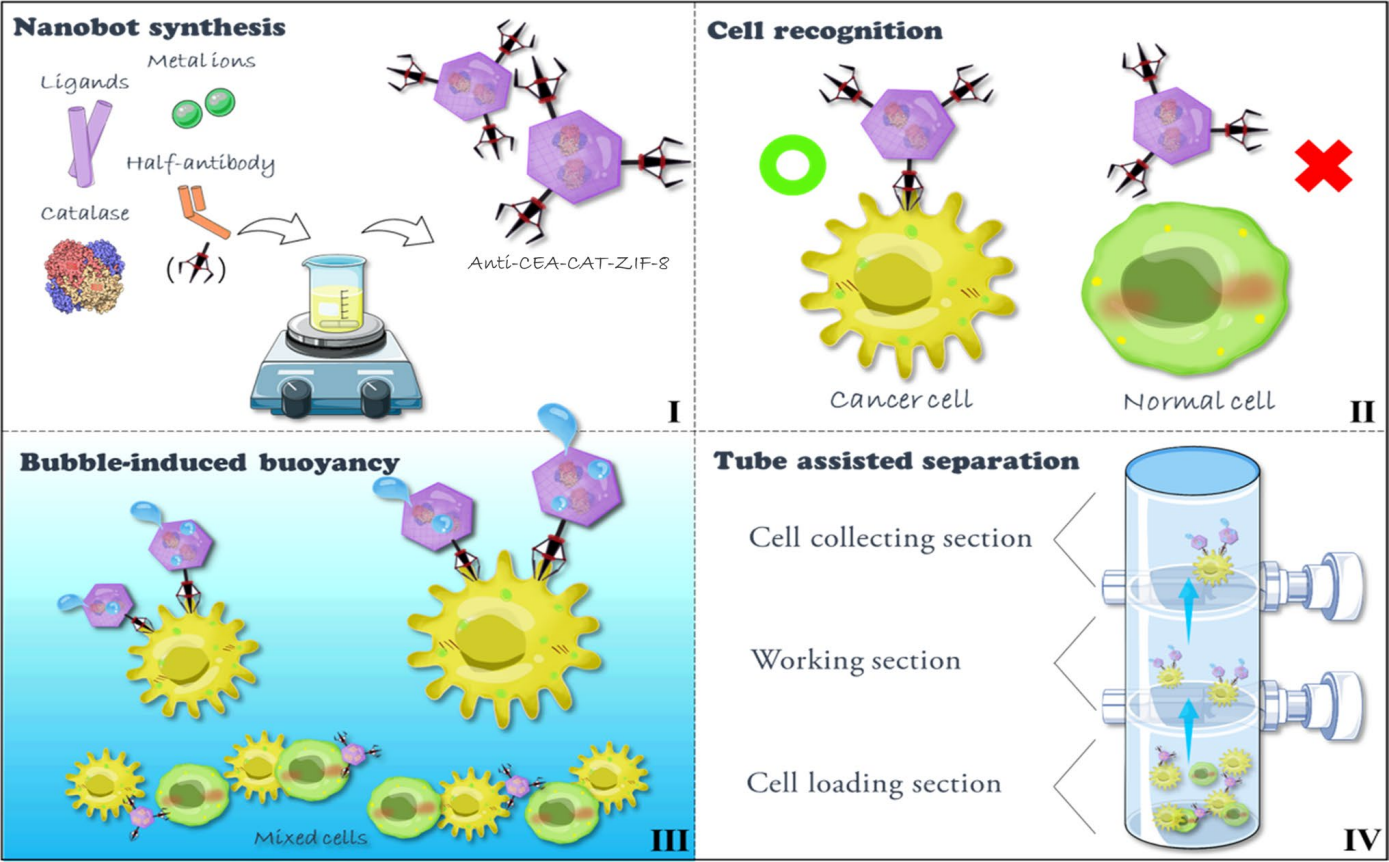
Schematic illustration of the anti-CEA-CAT-ZIF-8 nanobot synthesis and its autonomous cell “find-and-fetch” process with a customized glass column

Here, we report a biocatalytic buoyancy-driven nanobot system with specific antibody functionalization for efficient and autonomous cell recognition and enrichment from mixed cell lines. Zeolitic imidazolate framework-8 (ZIF-8), a typical type of metal–organic frameworks (MOFs) with zeolite topology, is considered as a biocompatible nanobot matrix, and the bioactive enzyme catalase (CAT) can be facilely incorporated to produce O_2_ gas bubbles from H_2_O_2_ and induce drastic buoyancy enhancement [30, 31, 54]. Considering the potential working environment, the catalytic properties of catalase retained in the presence of hemoglobin in blood has been reported before [38]. The ZIF-8 matrix can be easily degraded under mild acidic conditions or in the presence of biocompatible metal cheating agents (e.g., EDTA) for easy cell recovery. To endow the ZIF-8 nanobots with specific recognition capacity, monoclonal anti-CEA antibody was reduced to half fragments and modified on the surface of the nanobots via spontaneous Zn–S bonding [39–41]. To simplify the nanobot-enabled autonomous cell “find-and-fetch”, a customized glass column with two switches was designed (Scheme 1). It is the first time that buoyancy driven nanobots were applied in cell separation, which take the advantages of the long-distance sustainable gas bubble drag force. This nanobot system exhibited remarkable target cell recognition and transportation capabilities from mixed cell lines, highlighting its potential as an alternative tool for cell manipulation in the future.

## Experimental Section

### Materials

Zinc nitrate hexahydrate, 2-methylimidazole, catalase from bovine liver (CAT), fluorescein diacetate, BioTracker 655 Red Cytoplasmic Membrane Dye, bovine serum albumin (BSA), Tween 20, tris(2carboxyethyl)-phosphine (TCEP), Pluronic F-127 (PF-127), paraformaldehyde, and Triton X-100 were purchased from Sigma Aldrich. DMEM culture medium, fetal bovine serum, phosphate-buffered saline 1 × PBS buffer, antibody dilution buffer, penicillin/streptomycin, EDTA powder, Hoechst 33342 (nucleic acid stain) were purchased from Thermo Fisher Scientific. Anti-carcinoembryonic antigen–antibody (ab133633, Alexa Fluor® 488 ab214868), goat polyclonal secondary antibody to Rabbit IgG (Alexa Fluor® 647, ab150079), goat polyclonal secondary antibody to Rabbit IgG (Alexa Fluor® 488, ab150077), cell counting kit (CCK-8) were purchased from Abcam. Percoll gradients were purchased from TBD sciences (Tianjin, China). Reagents were used without any further purification. The particle movement was recorded as a video using a light microscope placed sideways. A thin glass chamber is designed by stacking two coverglass filled with solutions containing various amounts of H_2_O_2_, and the moving distance was pre-calibrated with a 4 mm stainless steel sphere. ImageJ software was used to extract the particle motion trajectory that enabled the calculation of the particle velocity change from the video.

### Buoyancy Force Calculation

The nanomotor was estimated to be a sphere with the radius of 250 nm.

$${F}_{buoyancy}={\rho }_{water}gV={10}^{3}kg/{m}^{3}\times 9.8N/kg\times \frac{4}{3}\pi {\left(2.5\times {10}^{-7}m\right)}^{3}=6.4\times {10}^{-16}N$$

### Synthesis and Structural Characterization

#### General Experimental Conditions

Confocal laser scanning fluorescence microscopy images were taken with an Olympus FV3000 Confocal laser scanning microscope. Scanning electron microscopy (SEM) images of samples were taken on an Apreo-S SEM. The fluorescence intensity was measured with CLARIOstar Plus microplate reader. Flow cytometry analysis was carried out with a BD LSRFortessa cell analyzer. The particle movement was recorded as a video using a light microscope. ImageJ software was used to extract the particle motion curve that enabled the calculation of the particle velocity change from the video.

#### Synthesis and Structural Characterization

*Fluorescent labeling of CAT*: CAT was fluorescently labeled with fluorescein isothiocyanate (FITC). CAT (40 mg) and FITC (1 mg) were dissolved in phosphate buffer (2.5 mL, pH 7.4, 0.5 M) and kept stirring for 2 h at room temperature. The labeled enzymes were purified and collected through a NAP-25 column (GE Healthcare).

*Synthesis of nanobot particles*: The synthesis of CAT@ZIF-8 followed a previous report by our group with slight modification [31]. Briefly, CAT (0.3 mg) was dissolved in 2-methylimidazole solution (800 µL, 860 mM) and zinc nitrate solution (200 µL, 45 mM) was then added quickly followed by continuous stirring for 1 h. The resultant particles were collected by centrifugation (Eppendorf Centrifuge 5418) at 5000 rpm for 2 min. The particles were washed with Milli-Q water and centrifuged at 5000 rpm for three times and finally resuspended in 1 mL Milli-Q water.

All the precursor solutions were filtered through a 0.22 μm pore size nitrocellulose membrane before use.

*Encapsulation efficiency*: The encapsulation efficiency of the FITC labeled CAT was tested with fluorescence spectrophotometry. A series of standard fluorescent CAT solution with gradient concentrations were prepared. The fluorescence intensity of each solution at 520 nm was measured with the microplate reader and the results were used as the standard curve.

The supernatant of the synthesized particles was measured and compared with the standard curve.

*Antibody labeling of nanobot particles*: The synthesis of the half-fragmented antibody followed literature from Jeon and co-workers with slight modification [41]. TCEP (12 μL, 1.4 mg mL^−1^) in PBS buffer was added to antibody dilution buffer (400 μL, final TCEP concentration 0.17 mM) with CEA antibody (12 μg). The solution was well mixed and incubated for 1 h at room temperature to reduce the disulfide bond between the heavy and light chains of the antibody. After incubation, the half-fragmented antibodies were mixed with centrifuged CAT@ZIF-8 nanoparticles (400 μL) and incubate for 1 h at room temperature with gentle mixing. The half-fragmented antibody was attached to the nanoparticles via spontaneous Zn–S bonding. The CEA antibody conjugated CAT@ZIF-8 (anti-CEA-CAT-ZIF-8) was collected by centrifugation and washed with antibody dilution buffer to get rid of the free antibody. The anti-CEA-CAT-ZIF-8 was then incubated alternately with PF-127 (0.02 wt%) and BSA (1%) in PBS buffer for half an hour to prevent nonspecific binding and washed with PBS buffer afterward.

*Cell culture*: The MCF-7 breast cancer cells and L929 fibroblast cells were cultured in Dulbecco's Modified Eagle Medium (DMEM) containing 10% fetal bovine serum and 1% penicillin streptomycin at 37 °C in a humidified environment containing 5% CO_2_. The cells were pre-cultured without further modification before the experiments.

To prepare suspended cells, the MCF-7 cell line and L929 cell line were washed twice with PBS and incubated in trypsin for 5 min. FBS was added to the plate to cease the enzymatic digestion. The detached cells were collected by centrifugation at 400 g for 5 min and washed three times with PBS to remove the extra trypsin.

To prepare attached cells on glass slides, the slides were cleaned and placed in 6-well cell culture plates. The suspended cells were counted and seeded in 6-well plates with the concentration of 20,000/well. The cells were cultured for 12 h before use.

*SEM characterization of specific binding between anti-CEA-CAT-ZIF-8 and cancer cells:* The round glass slides were placed into 12-well plates. MCF-7 cells were seeded on the glass slides in 12-well plates and cultured for 12 h before the incubation of anti-CEA-CAT-ZIF-8 at 4 °C for 1 h. The treated cells were washed with PBS buffer and fixed with 4% paraformaldehyde for 30 min. After fixation, the cells were treated by gradient dehydration with a series of ethanol solutions in the order of 50%, 70%, 90%, 100% concentrations for 15 min each. The cell samples were dried overnight before imaging.

*SDS-PAGE electrophoresis*: The SDS-PAGE of CEA antibodies and fragments was carried out following literature from Maquieira and co-workers with slight modification [52]. The assay was performed in 8% Bis–Tris acrylamide minigel. BeyoColor™ prestained color protein marker (10–170 kD) was used for the ladder standard. Whole antibodies (8 μg) and antibody fragments were suspended in PBS (40 μL) with SDS sample loading buffer (10 μL). Each sample was loaded to a well and the gels were run at 20 mA for 45 min and 30 mA for 20 min. After electrophoresis, the gel was dyed with Coomassie Brilliant Blue solution at room temperature overnight and detained with ethanol/acetic acid solution (5%/10%) till excess staining was removed.

*Immunoassay*: The round glass slides were placed into 12-well plates. MCF-7 cells and L929 cells were seeded on the glass slides in 12-well plates and cultured for 12 h before the experiment. The cells were fixed with 4% paraformaldehyde for 30 min and washed with PBS buffer for three times. The cells were incubated with Triton X-100 (0.1%) and BSA (3%) for 20 and 30 min, respectively. The Triton X-100 was used to increase the permeabilization and BSA was used to block the non-specific binding. After the pretreatment, the two cell lines were incubated with CEA mAb at 4 °C overnight, followed by washing with PBS for three times and incubating with fluorescent secondary antibody for 1 h. The cell nuclei were stained with Hoechst 33342 and the images were taken under a fluorescence microscope.

*Antibody loading efficiency*: To measure the loading efficiency of the half antibody, the absorbance of the supernatant from synthesized anti-CEA-CAT-ZIF-8 was measured at 310 nm with Nanodrop and compared with the standard half antibody solution.

*Cell recognition and isolation with attached cells*: The MCF-7 breast cancer cells and L929 fibroblast cells were cultured following the attached cells protocol. The MCF-7 cell line was stained with Hoechst (blue) to distinguish from the normal L929 cell line. Both of the cell slides were washed with PBS buffer solution and transferred to a same well followed by incubation with 3% BSA for 30 min to minimize non-specific binding. 5 μL of anti-CEA-CAT-ZIF-8 was added to the well in dark, followed by gentle shaking for 2 h at 4 °C. The extra anti-CEA-CAT-ZIF-8 was washed out with PBS buffer solution gently after incubation. The treated cells were detached from the slides with trypsin and transferred to the bottom section of the customized glass column. The column was washed with PBS twice after the cell addition and H_2_O_2_ solution in PBS buffer was added to fill the upper sections. The lower switch was turned on to expose the cells to H_2_O_2_, and the recognized cancer cells were carried with the mobile anti-CEA-CAT-ZIF-8 to the upper section. Both of the switches were turned off after 10 min and the isolated cells were transferred out separately. To identify the isolation efficiency, the isolated cells on top layers were taken for flow cytometry counting.

*Cell recognition and isolation with suspended cells*: The MCF-7 cell line and L929 cell line were cultured following the detached protocol and the MCF-7 cell line was stained with Hoechst (blue) to distinguish from the normal L929 cell line. Both of the cells were washed with PBS buffer and detached from the petri dish with trypsin. Both of the cell suspensions were adjusted to an equal number of 10,000 before mixing and different amount of anti-CEA-CAT-ZIF-8 were added in dark, followed by gentle shaking every 30 min for 2 h at 4 °C. The treated cells were transferred to the bottom section of the customized glass column and treated with similar methods as above.

The human blood monocytes were isolated and purified by density gradient centrifugation with Percoll kit (TBD, Tianjin, China) according to the official instruction. Briefly, heparin was added as the anticoagulant and the treated blood was diluted with commercially available diluent and mixed well with ficoll. The mixture was centrifuged at 1350 rpm and the second layer of the four layers was transferred to another centrifuge tube. The monocytes were washed with PBS and centrifuged at 850 rpm before usage.

*Cell viability assays*: The cells recovered from the cell collecting section were incubated in trypsin with EDTA (50 mM) for 2 min and washed with FBS and PBS buffer. The incubation and washing process were repeated once. The collected cells were counted and cultured in a 96-well plate in Dulbecco's Modified Eagle Medium (DMEM) containing 10% fetal bovine serum and 1% penicillin streptomycin at 37 °C overnight (approximately 16 h) before CCK-8 test. The CCK-8 solution was added to the cells with no pre-mixing and the absorbance of each well was measured at 460 nm with a micro plate reader after incubation for 4 h.

## Results and Discussion

### Characterization and Motion Analysis of Anti-CEA-CAT-ZIF-8 Nanobots

To construct the biocatalytic nanobots, CAT was incorporated into ZIF-8 (CAT-ZIF-8) via a facile one-step biomineralization process [42, 43]. An anti-carcinoembryonic antigen (CEA) antibody was then used to functionalize the nanobots with specific cell recognition ability [44, 45]. CEA antigens are highly overexpressed on several common cancer cells including colorectal [46, 47], pancreatic [48, 49], and gastric cancer cells [50]. Due to its tumor-associated expression, CEA has become a crucial biomarker in cancer diagnostics, providing valuable insights into disease detection, prognosis, and treatment response assessment. Its clinical significance has led to extensive research aimed at harnessing CEA as a target for novel therapeutic strategies in oncology [51]. The resulted anti-CEA-CAT-ZIF-8 nanobots were further incubated with PF-127 and BSA solution to prevent non-specific binding (see Supporting Information for detailed synthesis procedure).

**Fig. 1:**
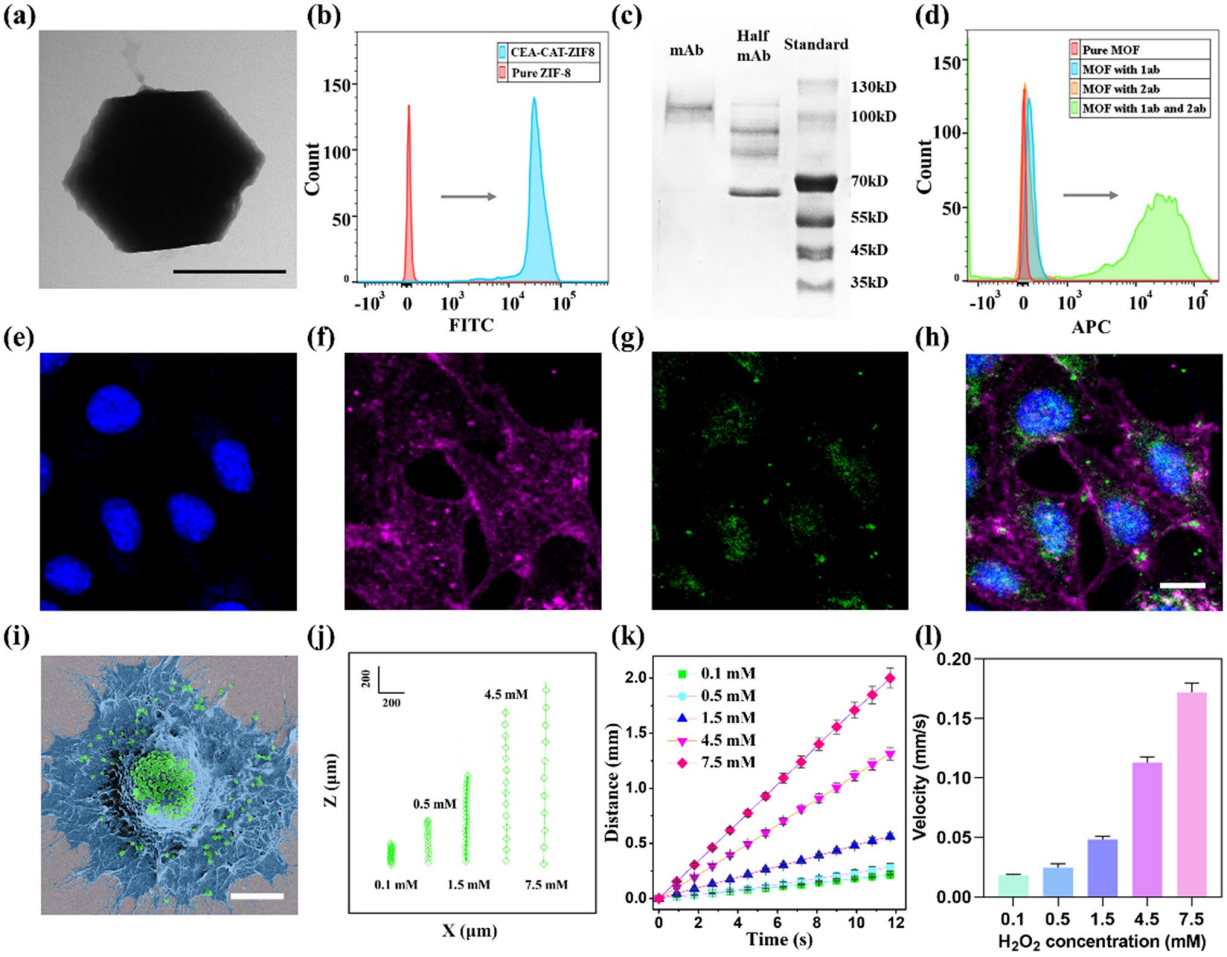
Characterization of anti-CEA-CAT-ZIF-8 nanobots and motion analysis. **a** TEM image of the anti-CEA-CAT-ZIF-8 nanobots (scale bar 500 nm). **b** Flow cytometry results of pure ZIF-8 and anti-CEA-CAT-ZIF-8 in histogram. **c** SDS-PAGE of CEA antibody in the reduced and non-reduced forms. A standard ladder was used to locate the position of the bands. **d** Flow cytometry results of pure ZIF-8, anti-CEA-CAT-ZIF-8, anti-CEA-CAT-ZIF-8 with primary antibody, and anti-CEA-CAT-ZIF-8 with both primary and secondary antibodies. **e–h** Confocal laser scanning fluorescence microscopy images. The cell nuclei were stained with Hoechst (blue), the cell membranes were stained with BioTracker 655 red cytoplasmic membrane dyes (magenta), and the anti-CEA-CAT-ZIF-8 was labeled with FITC (green). **h** The merged image (Scale bar 10 μm). **i** Representative SEM image of anti-CEA-CAT-ZIF-8 with MCF-7 cell (Scale bar 5 μm). The cell is pseudocolored in blue and anti-CEA-CAT-ZIF-8 is pseudocolored in green. **j** Representative nanobot-cell trajectories with different concentrations of H_2_O_2_. **k** The moving distance and **l** ascending velocity of the nanobot-cells. The error bars represent the standard deviation for three independently recorded trajectory events, and at least 50 particles were analyzed in each event

The SEM and transmission electron microscopy (TEM) images of the anti-CEA-CAT-ZIF-8 revealed the nanobot with a rhombic dodecahedral morphology around 500 nm in diameter (Figs. 1 a and S1), which were similar to the standard ZIF-8 nanocrystals. To confirm the successful encapsulation of CAT, the enzyme was labeled with fluorescein isothiocyanate (FITC) in advance, and the fluorescence intensity of the synthesized anti-CEA-CAT-ZIF-8 was measured with flow cytometry. The results clearly demonstrate an increased fluorescence signal of CAT (Figs. 1 b and S2), indicating the successful encapsulation of CAT in the nanobot.

The loading efficiency of the FITC-labeled enzyme was measured to be ~ 95% by measuring the fluorescence of the supernatant of the synthesis solution (Fig. S3). Powder X-ray diffraction (XRD) patterns of pure ZIF-8 and anti-CEA-CAT-ZIF-8 were provided in Fig. S4, indicating the preservation of the ZIF-8 crystal structure after enzyme incorporation and antibody functionalization. To study the storage stability of the anti-CEA-CAT-ZIF-8 in PBS buffer, the nanobots were immersed in PBS for 24 h and recollected by centrifugation for SEM characterization. The retained morphology of the immersed nanobots was illustrated in Fig. S5, indicating the nanobots retained their structure integrity after incubation. To prove the specific targeting ability of the CEA antibody to cancer cells, the CEA-overexpressing MCF-7 cell line and CEA negative L929 cell line were tested with an in vitro immunofluorescence assay. The MCF-7 cell line is a breast cancer cell model that exhibits higher levels of carcinoembryonic antigen, a protein often associated with tumor progression. In contrast, the CEA-negative L929 cell line lacks CEA expression, making it a valuable tool for studying cellular processes independent of CEA-related effects and providing insights into non-cancerous cellular behavior. The cells were incubated with primary antibody (rabbit anti-CEA IgG) and Alexa Fluor 488 labeled secondary antibody (goat anti-rabbit IgG, green). The cell nuclei were stained with Hoechst (blue). The MCF-7 cells exhibited distinct green fluorescence around the nuclei (Figs. S6a and S4b, c), while in negative control the L929 cell line exhibited neglectable green fluorescence (Figs. S6d and S4e, f), indicating the specific targeting capacity of the anti-CEA antibody on the MCF-7 cell line. SDS-PAGE was employed to confirm the successful production of half antibody using TCEP as the reducing agent (Fig. 1c) [52]. The whole antibodies and antibody fragments were separated by electrophoresis and dyed with Coomassie Brilliant Blue solution. According to the standard ladder, the results showed mainly the expected bands between 130 and 100 kDa for the whole antibody, and 70 kDa for the half antibody fragments. Flow cytometry was also applied to confirm the nanobot surface modification with half antibody (Fig. 1d). The fluorescence intensity of anti-CEA-CAT-ZIF-8 incubated with goat anti-rabbit secondary antibody (Alexa Fluor 647 labeled) was compared with anti-CEA-CAT-ZIF-8, indicating the successful binding to the primary antibody on the nanobot surface. To rule out the interference of nonspecific binding, pure ZIF-8 was also incubated with the secondary antibody, which indicated neglectable fluorescence alteration compared to the pure ZIF-8. The loading efficiency of the half antibody was calculated to be 40% by measuring the absorbance of the supernatant from the anti-CEA-CAT-ZIF-8 synthesis solution at 310 nm (Fig. S7). The zeta potential measurements showed distinctive charge differences upon CAT loading and CEA antibody functionalization (Fig. S8). Collectively, the above results confirmed the successful conjugation of functional half-antibody fragments to the nanobot surface [53].

To assess the cell binding capacity of anti-CEA-CAT-ZIF-8 nanobots, the FITC-labeled nanobots were incubated with MCF-7 cells. The treated cells were detached afterward and measured with flow cytometry (Fig. S9). According to the results, the cells with anti-CEA-CAT-ZIF-8 exhibited higher fluorescence intensity compared to the non-CEA functionalized CAT-ZIF-8, indicating the enhanced specific cell binding capacity of the CEA antibody functionalized nanobots. The peak of the non-CEA functionalized CAT-ZIF-8 group was ascribed to the non-specific binding of the nanobots. Confocal laser scanning fluorescence microscopy (CLSM) and SEM images further verified the attachment of anti-CEA-CAT-ZIF-8 to the MCF-7 cells (Fig. 1e-i). Imaging flow cytometry was further employed to quantify the nanobot-cell attachment (Fig. S10), which suggested that on average of 100 nanobots were attached to each MCF-7 cell.

When the anti-CEA-CAT-ZIF-8 nanobots were exposed to low concentrations of H_2_O_2_, the encapsulated CAT started instant nano-bubble formation, which was strongly bound to the hydrophobic ZIF-8 framework. The attached oxygen bubbles altered its buoyancy, resulting in self-propelled vertical motion [30, 31]. The oxygen increment in the working environment was measured (Fig. S11), indicating the limited oxygen amount dissolved in the solution.

The vertical motion of the nanobots was recorded with an optical microscope coupled with a high-resolution camera and the trajectories were extracted with Image J. To calculate the correlation between the H_2_O_2_ concentration and the nanobot velocity, the travel distance with fixed time interval $\Delta t$ was calculated according to Eq. (1):

1 $${\left.\Delta x\right|}_{\Delta t}={\left({\left({x}_{\Delta t}-x\right)}^{2}+{\left({y}_{\Delta t}-y\right)}^{2}\right)}^{1/2}$$

where ${x}_{\Delta t}$ and ${y}_{\Delta t}$ are the coordinates of the particle in the plane of motion after time interval $\Delta t$. The moving distance and ascending velocity of the anti-CEA-CAT-ZIF-8 nanobots with different amounts of H_2_O_2_ were shown in Figs. S12 and S13. The buoyancy force of the individual nanobot ($6.4\times {10}^{-16}N)$ was estimated according to Eq. (2), assuming each nanobot as a sphere with the radius of 250 nm.

2 $${F}_{\mathrm{buoyancy}}={\rho }_{\mathrm{water}}gV$$

In addition, our previous work has both experimentally and computationally verified the individual and collective dynamic motion behaviors of these buoyancy-driven nanobots [54].

To study the capability of nanobots in cell transportation, anti-CEA-CAT-ZIF-8 was incubated with CEA-positive MCF-7 cells in the presence of low amount of H_2_O_2_ ranging from 0.1 to 7.5 mM, and their motion behavior of the nanoparticles attached to cancer cells was recorded. The cells were fixed with 4% paraformaldehyde after detachment to maintain their morphology. The vertical motion of the nanobot-cell hybrids was recorded and analyzed with a high-speed camera (Fig. 1j). The results suggest that the ascending velocity of the anti-CEA-CAT-ZIF-8 with MCF-7 was positively correlated with the concentration of the H_2_O_2_ fuel (Fig. 1k, l). Based on these results, it was determined that the nanobots achieve sufficient buoyancy, rapidly ascending to the surface of the solution (depth ~ 4 cm) within the glass column, with times ranging from approximately 1800 s (30 min) at the lowest H_2_O_2_ concentration of 0.1 mM to 240 s (4 min) at the highest H_2_O_2_ concentration of 7.5 mM. Consequently, subsequent cell separation experiments were conducted at a H_2_O_2_ concentration of 1.6 mM for 15 min to ensure efficient bubble-induced buoyancy while preventing free bubble detachment, which could disrupt the nanobots’ vertical motion [54].

### Anti-CEA-CAT-ZIF-8 Nanobots on Cell Recognition and Autonomous Separation with Adherent Cells

To evaluate the potential toxicity of anti-CEA-CAT-ZIF-8 and H_2_O_2_ on the cells, different amounts of anti-CEA-CAT-ZIF-8 ranging from 0.2 to 4 μL were applied to both MCF-7 and L929 cells for a simulated nanobot cell separation period of 15 min followed by cell viability measurement with cell counting kit-8 (CCK-8). Results suggested that the cells maintained a high viability over 80% with up to 2 μL of nanobots added (Fig. S14). We then assessed the cell viability in the presence of H_2_O_2_ ranging from 0.2 to 6.4 mM with a fixed amount (0.5 μL) of nanobots for the same period (Fig. S15), and the results suggested that the cell viability remained over 80% at up to 1.6 mM H_2_O_2_ for both cell lines. Therefore, a total amount of 1.6 mM H_2_O_2_ was applied in the following cell separation experiments to ensure efficient nanobot mobility without impacting the cell viability.

**Fig. 2:**
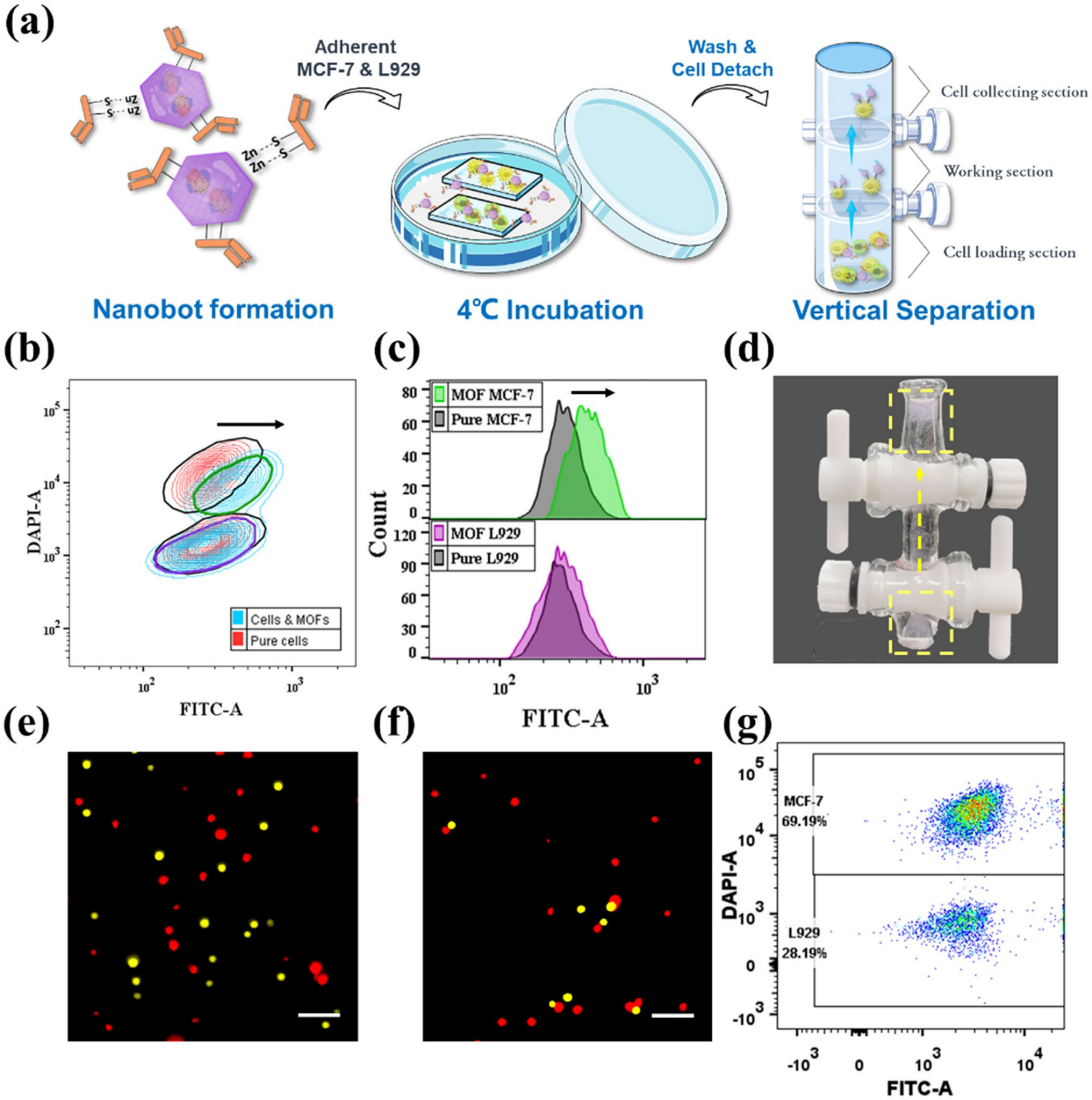
Nanobot-enabled cell recognition and separation with adherent cells. **a** Schematic illustration of the experiment. Both of the cells were transferred together in a well and incubated with FITC labeled anti-CEA-CAT-ZIF-8. The mixed cell sample was transferred to the bottom layer of the customized glass column for vertical isolation. **b** Contour plot and **c** Spectra from flow cytometry. MCF-7 was stained with Hoechst (upper green contour). The results from the initial control sample (black contour and black spectrum) and treated cells were stacked together for comparison. **d** The screenshot of customized glass tube with nanobots. The position of the nanobots (stained with rhodamine) was indicated with the yellow dash line. The path of the motion was indicated with a yellow arrow. Fluorescence microscopy images of **e** control and **f** separated cell samples. The MCF-7 (pseudocolored in red) and L929 (pseudocolored in yellow) were dyed with Hoechst and calcein AM, respectively (Scale bar 200 μm). **g** Dot plot of segregated cell sample from flow cytometry

We first assessed the performance of the anti-CEA-CAT-ZIF-8 nanobots in cell recognition and autonomous separation using adherent cells (Fig. 2a). Both CEA-positive MCF-7 and CEA-negative L929 cell lines were seeded on glass slides in a 12-well plate with the same initial cell count and cultured overnight. Prior to the addition of the nanobots, the cells were pretreated with 1% BSA to block the non-specific binding and the MCF-7 were stained with Hoechst (blue) to distinguish from L929 using flow cytometry. After that, both cell lines were transferred to the same well and incubated with FITC labeled anti-CEA-CAT-ZIF-8 nanobots accompanied by continuous shaking. After an incubation period of 2 h, the cells were detached from the glass slides and analyzed using flow cytometry (Fig. 2b, c). Based on the stained nuclei of MCF-7, the green contour with higher blue fluorescent intensity was considered as MCF-7. According to the flow cytometry results, the fluorescence intensity of FITC barely changed in the L929 population, indicating neglectable binding of anti-CEA-CAT-ZIF-8 nanobots to the L929 cells. In contrast, most of the MCF-7 cells appeared to be associated with the nanobots. To realize the autonomous cell transportation/separation, a customized glass column was applied (Figs. 2d and S16). The mixed cell sample was detached from the glass slide and then transferred to the cell loading section in the glass column and separated from H_2_O_2_ (1.6 mM) in the working section (Fig. 2a). The loaded cells were exposed to the H_2_O_2_ fuel by turning on the bottom switch, and the nanobot ascending motion was enabled by the biocatalytic bubble formation in the presence of H_2_O_2_, which autonomously carried the antibody-bound target cells to the top cell collecting section within 15 min. The top switch was then turned off to isolate the floated cells in the collecting section (see Video S1).

Fluorescence microscopy images of the recovered cells after the nanobot-enabled separation showed MCF-7 cells in dominance compared to the starting 1:1 cell mixture before separation (Figs. 2 e, f and S17). Flow cytometry was then employed to qualitatively assess the cell separation efficiency (Fig. 2 g). Compared with the starting cell mixture of 1:1 MCF-7:L929 ratio, the ratio of the recovered cells by nanobots increased to 2.45:1, showcasing appreciable cell separation efficiency. Remarkably, the recovery efficiency of MCF-7 cells reached over 99%. The presence of L929 cells in the final cell population were ascribed to the non-specific binding of anti-CEA-CAT-ZIF-8. Overall, the results proved that the nanobots exhibited great cell recognition and separation efficiency, however, the working conditions for future clinical diagnosis normally require suspended cells. To further exploit the practicability and autonomous potential of the nanobots in cell isolation, we also investigated the cell separation performance directly with suspended cells.

### Anti-CEA-CAT-ZIF-8 Nanobots on Cell Recognition and Autonomous Separation with Suspended Cells

**Fig. 3:**
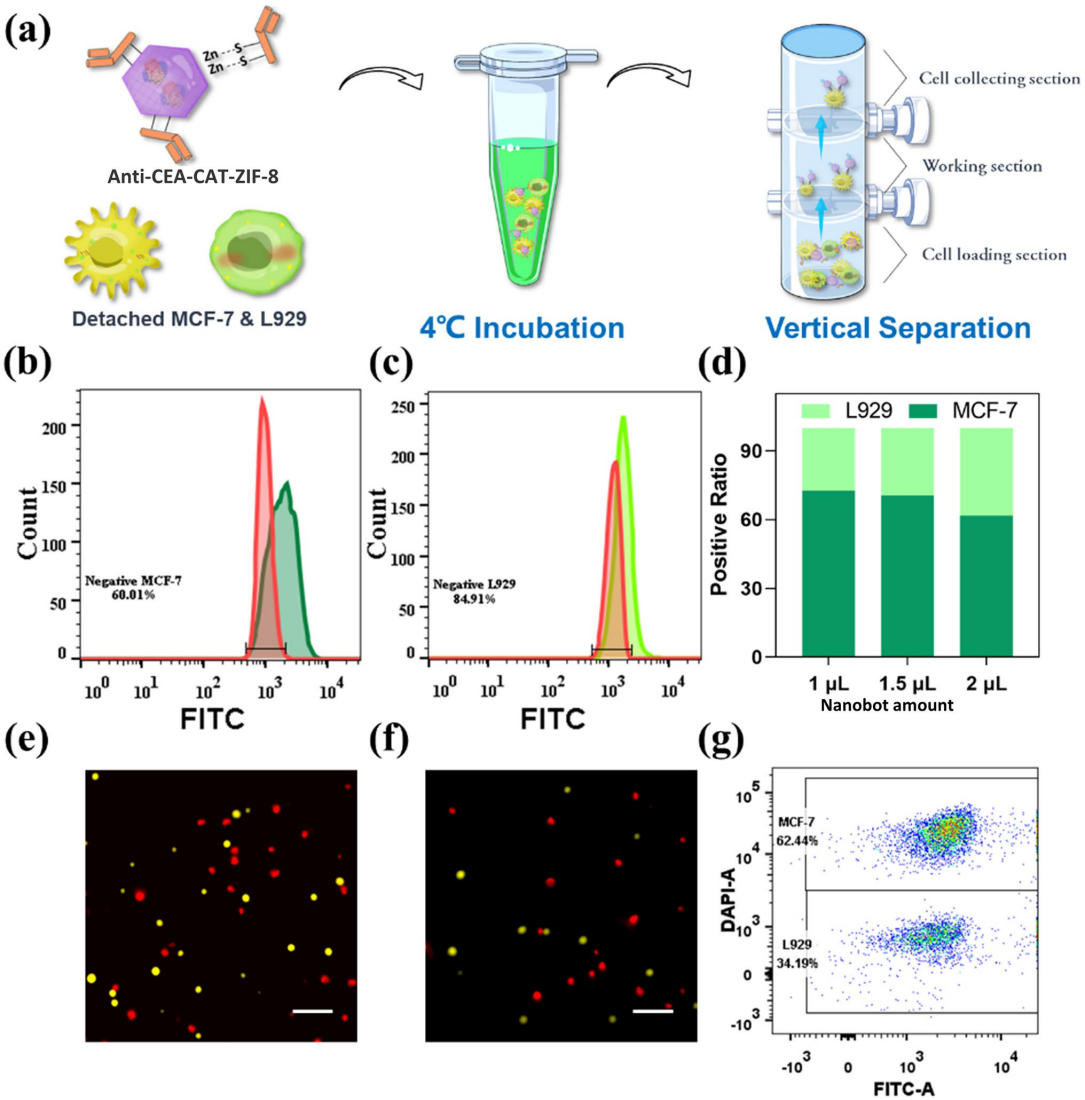
**a** Schematic illustration of the nanobot-enabled autonomous cell recognition and separation with suspended cells. The spectrum shift of **b** MCF-7 and **c** L929 with 1 μL of FITC-labeled anti-CEA-CAT-ZIF-8. The peaks of pure cells were displayed as red. **d** Stacked bar of the nanobots associated cells calculated from the flow results with 1, 1.5, and 2 μL of anti-CEA-CAT-ZIF-8. (e–f) Fluorescence images of **e** control and **f** segregated cell samples. The MCF-7 (pseudocolored in red) and L929 (pseudocolored in yellow) were dyed with Hoechst and calcein AM, respectively (Scale bar is 200 μm). **g** Dot plot of segregated cell sample from flow cytometry

Cell recognition and separation process using suspended cells were illustrated in Fig. 3 a. Prior to the experiment, the cells were pretreated with 1% BSA to block the non-specific binding. The cells were washed with PBS buffer and lifted from the petri dish using trypsin. Prior to the addition of the nanobots, both MCF-7 and L929 cell suspensions were mixed at an equal amount of 10,000 cells per cell line and the FITC labeled anti-CEA-CAT-ZIF-8 were added to the cells in dark, followed by gentle shaking every 30 min for 2 h at 4 °C. Different from the cells in adherent status, the excess of nanobots applied to the cell suspension mixture could not be washed out, therefore, determining the appropriate amount of the nanobots to be added is essential. Excess amount of nanobots could increase non-specific binding, which will negatively impact the specificity in cell targeting and separation. Accordingly, the targeting specificity of different amounts of anti-CEA-CAT-ZIF-8 nanobots ranging from 1 to 2 μL were studied (Fig. S18). The results indicated that the amount of nanobots attached to both cell lines increased with increasing amount of added anti-CEA-CAT-ZIF-8. Next, flow cytometry was employed to quantitatively study the cell binding specificity using suspended cell mixtures. The percentage of MCF-7 cells associated with nanobots (40%) was higher compared to L929 cells (15.1%), indicating the specific binding capacity of anti-CEA-CAT-ZIF-8 to MCF-7 (Fig. 3 b, c). The ratios of both cell lines associated with anti-CEA-CAT-ZIF-8 were collected from the spectrums and displayed with stacked graph in Fig. 3 d, which indicates the increase in targeting specificity of MCF-7 with the less amount of anti-CEA-CAT-ZIF-8. To rule out the intrinsic differences in non-specific binding of nanobots between MCF-7 and L929, each cell line was separately incubated with 1, 1.5, and 2 μL of nanobots without CEA antibody functionalization. Flow cytometry analysis exhibited negligible differences in non-specific binding between these two cell lines (Fig. S19). Notably, introducing higher amount of nanobots is expected to increase the vertical drag force for cell separation, but could also induce significant non-specific binding to the unwanted cells. Therefore, the addition of 1 μL anti-CEA-CAT-ZIF-8 nanobots was applied in the following autonomous cell isolation experiment.

The mixed cell (MCF-7:L929 1:1) suspension in PBS containing the nanobots and H_2_O_2_ (1.6 mM) was transferred to the cell loading section of the customized glass column to initiate the autonomous cell transportation. After 15 min, the cells that were transported to the top collecting section were collected and analyzed (Fig. 3a). Fluorescence microscopy images of the recovered cells after nanobot-enabled separation showed significantly more MCF-7 cells presenting compared to the starting cell mixture before separation (Figs. 3e, f and S20). According to the flow cytometry results (Fig. 3g), the ratio between MCF-7 to L929 was calculated to be 1.83:1, indicating the successful isolation and concentration of CEA-positive MCF-7 cells from mixed cell population.

Although both the separation process with adherent cells and suspended cells showed acceptable recognizing efficiency, the specificity was limited by the non-specific binding of the nanobot to the cells. The limitation of the proposed nanobot system may be improved by surface modification in future work. Compared to the separation process with adherent cell model, the proposed process with suspended cells exhibited lower recognizing ratio of the target cells. The washing process with adherent cells ensured the saturated amount of nanobots in the incubation process and the removal of the non-specific binding afterward. However, the cell recognition with suspended cells requires simpler and more facile working process, which exhibited great potential in future development. The sacrificed specificity was expected to be resolved by the careful selection of different targeting molecules or further surface modification on the nanobot. Due to the low concentration of cancer cells in body fluids, we further increased the ratio of L929 to MCF-7 cells from 1:1 to 1000:1. When used at this ratio, it was estimated to be exposed to only 50 MCF-7 cells, and MCF-7 cells were still able to be captured (Fig. S21), suggesting its high capturing sensitivity.

To further enhance assay portability, we further tested the performance of the nanobot cell separation assay using a disposable syringe attached to a three-way valve instead of the glass column (Fig. S22). According to the flow cytometry results (Fig. S23), the ratio between positive to negative cells after nanobot cell separation assay was calculated to be 88.5:11.5% (cells were initially mixed at 50%:50% ratio), indicating the successful isolation and concentration of target cells from mixed cell population.

After cell enrichment, keeping the cells’ native state without negative impact from either the separation process or the bound nanoparticles is crucial in studying their true phenotypical and metastatic potential. Accordingly, the proliferation potential of the recovered cells was assessed. After obtaining the cells from the cell collecting section of the glass column, EDTA was introduced to degrade the attached nanobots and the cells were seeded in 96-well cell culture plates. SEM images of the seeded MCF-7 cells reveal their characteristic cobblestone morphology, firmly adhered to the surface, thus confirming their expected behavior (Fig. S24). After 16 h, the cell viability was assessed using CCK-8 in comparison with the untreated cells. The results demonstrated negligible impact on the viability of cells recovered from the nanobot-enabled autonomous cell isolation process (Fig. S25).

## Conclusions

In this work, we developed an antibody functionalized, biocatalytic MOF nanobot with remarkable power output capable of specific cell recognition and collective autonomous transportation from a mixed cell population. The biocatalytically generated O_2_ gas bubbles from H_2_O_2_ were preferentially retained by the hydrophobic nanobot matrix in aqueous environments, which produced enough buoyancy to effectively drive the antibody-bound cells upward. Unlike the conventional magnetic beads, the nanobots could be easily degraded and the recovered cells maintained their full proliferation potential. Although the low level of H_2_O_2_ used in this study shows minimal impact on the cell viability, future research directions in nanobot formulation could exploit alternative and more biocompatible chemical fuels, such as glucose through coupled glucose oxidase-catalase enzymatic cascade reaction or urea via urease biocatalysis. While attaching more nanobots to the cells could enhance the vertical drag force for cell separation, it comes at the cost of increased non-specific binding to unwanted cells. This issue could be addressed by implementing additional nanobot surface functionalization with antifouling molecules, such as polyethylene glycol, to minimize non-specific interactions. Nevertheless, the simplicity and versatility of the nanobot-assisted cell recognition and isolation offer a novel tool for diverse biomedical applications, highlighting foreseeable clinical and commercial opportunity.

### Supplementary Information

Below is the link to the electronic supplementary material.

Supplementary file1 (MP4 21893 KB)

Supplementary file2 (PDF 1538 KB)
