# Supplementary material for: Biocatalytic Buoyancy-Driven Nanobots for Autonomous Cell Recognition and Enrichment
Source: Nanomicro Lett. 2023 Oct 24;15:236. doi: 10.1007/s40820-023-01207-1 (PMC10597912; doi:10.1007/s40820-023-01207-1)
Supplement: Supplementary file 2 — Supplementary file2 (PDF 1538 KB) [file 40820_2023_1207_MOESM2_ESM.pdf]

Supporting Information for

Biocatalytic Buoyancy-Driven Nanobots for Autonomous Cell Recognition and Enrichment

Ziyi Guo[1, 2], Chenchen Zhuang[3], Yihang Song[2], Joel Yong[1], Yi Li[4, *], Zhong Guo[2, *], Biao Kong[5], John M. Whitelock[6], Joseph Wang[7], Kang Liang[1, 6, *]

[1] School of Chemical Engineering, Australian Centre for NanoMedicine, The University of New South Wales, Sydney, NSW 2052, Australia

[2] Medical College, Northwest Minzu University, Lanzhou 730000, P. R. China

[3] General Intensive Care Unit, Second Affiliated Hospital of Zhejiang University School of Medicine, Hangzhou, P. R. China

[4] School/Hospital of stomatology, Lanzhou University, Lanzhou 730000, P. R. China

[5] Department of Chemistry, Shanghai Key Lab of Molecular Catalysis and Innovative Materials, Collaborative Innovation Center of Chemistry for Energy Materials, Fudan University, Shanghai 200438, P. R. China

[6] Graduate School of Biomedical Engineering, The University of New South Wales, Sydney, NSW 2052, Australia

[7] Department of Nanoengineering, University of California San Diego, La Jolla, CA, 92093, USA

*Corresponding authors. E-mails: kang.liang@unsw.edu.au (K. L.), yxgz@xbmu.edu.cn (Z. G), yil@lzu.edu.cn (Y. L.)

Supplementary Figures

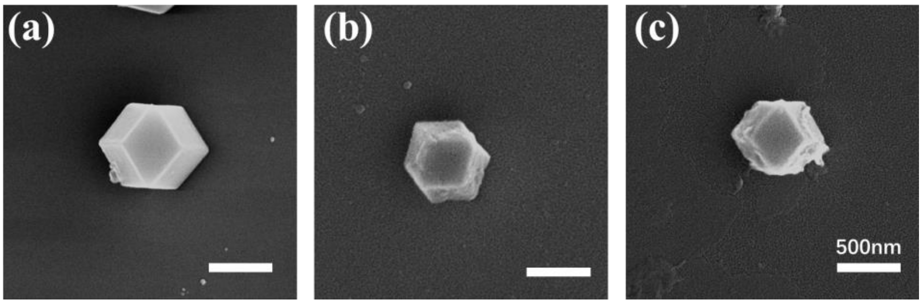

Fig. S1 SEM images of (**a**) pure ZIF-8, (**b**) CAT-ZIF-8, and (**c**) Anti-CEA-CAT-ZIF-8

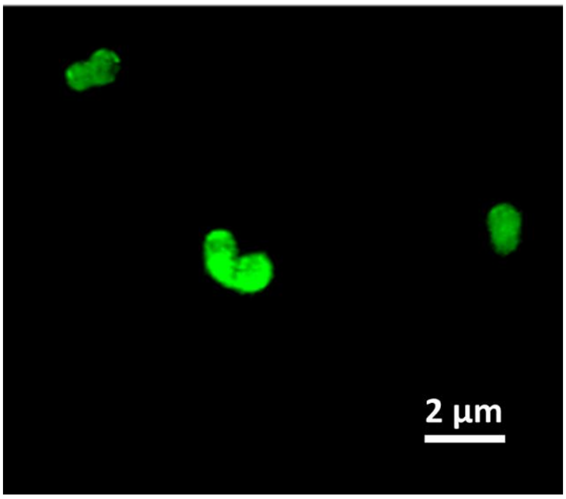

Fig. S2 CLSM image of FITC-CAT-ZIF-8

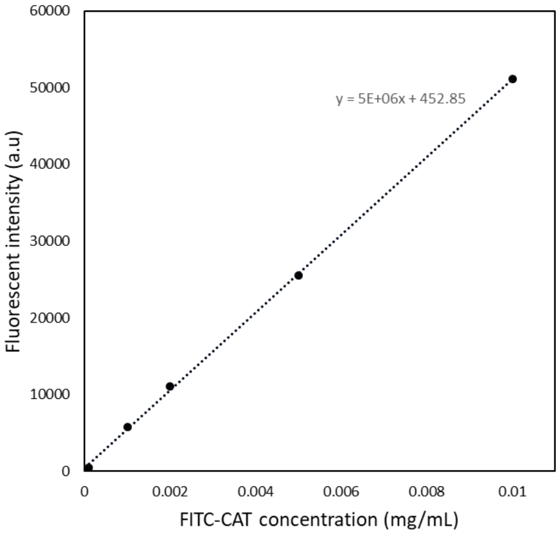

Fig. S3 Fluorescence intensity calibration curve of FITC-CAT

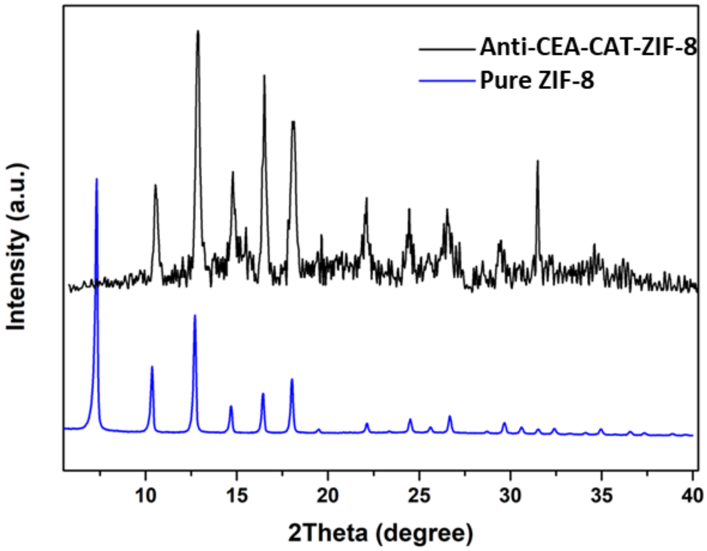

Fig. S4 Powder X-ray diffraction patterns of pure ZIF-8 and Anti-CEA-CAT-ZIF-8

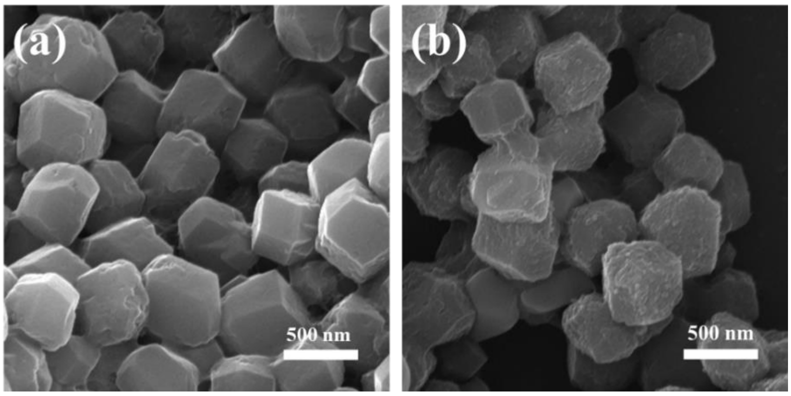

Fig. S5 SEM images of Anti-CEA-CAT-ZIF-8 after (**a**) 12 h and (**b**) 24 h incubation in PBS buffer solution

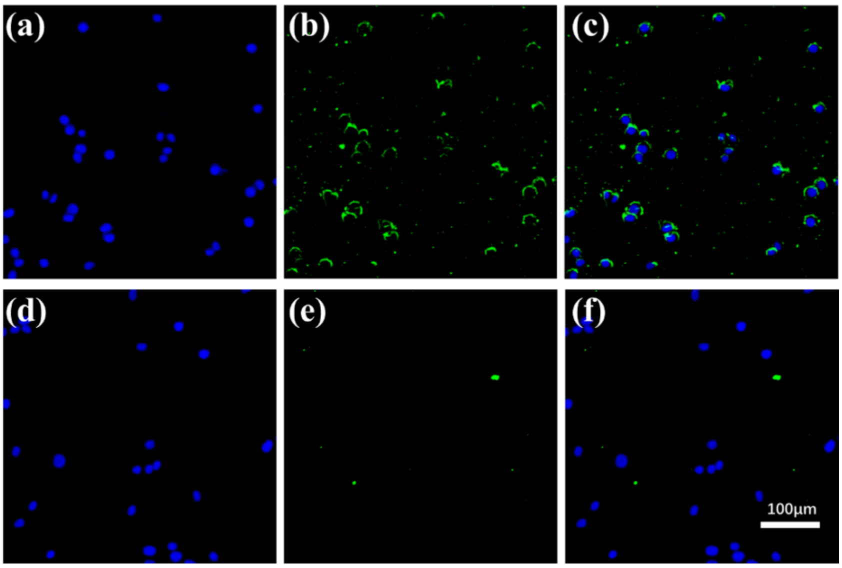

Fig. S6 Immunofluorescence images of (**a-c**) CEA positive MCF-7 cell line and (**d-f**) CEA negative L929 cell line. The secondary antibody was labeled with Alexa Fluor 488 (green). The cell nuclei were stained with Hoechst (blue)

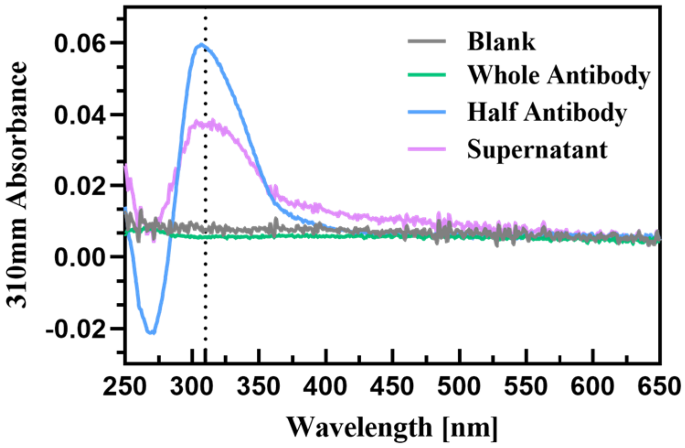

Fig. S7 The absorbance of the supernatant from synthesized Anti-CEA-CAT-ZIF-8 at 310 nm

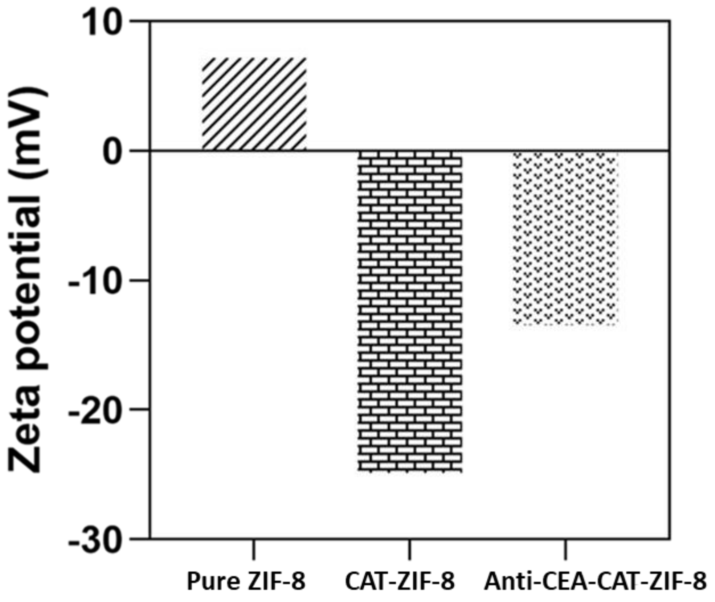

Fig. S8 Zeta potentials of pure ZIF-8, CAT-ZIF8, and AbCEA-CAT-ZIF8

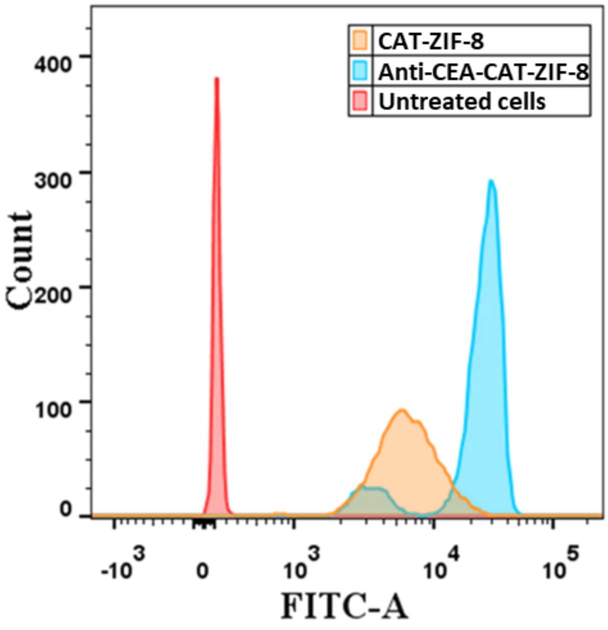

Fig. S9 Flow cytometry results of MCF-7 cells treated with FITC labeled CAT-ZIF-8 and Anti-CEA-CAT-ZIF-8

(a)
(b)
(c)

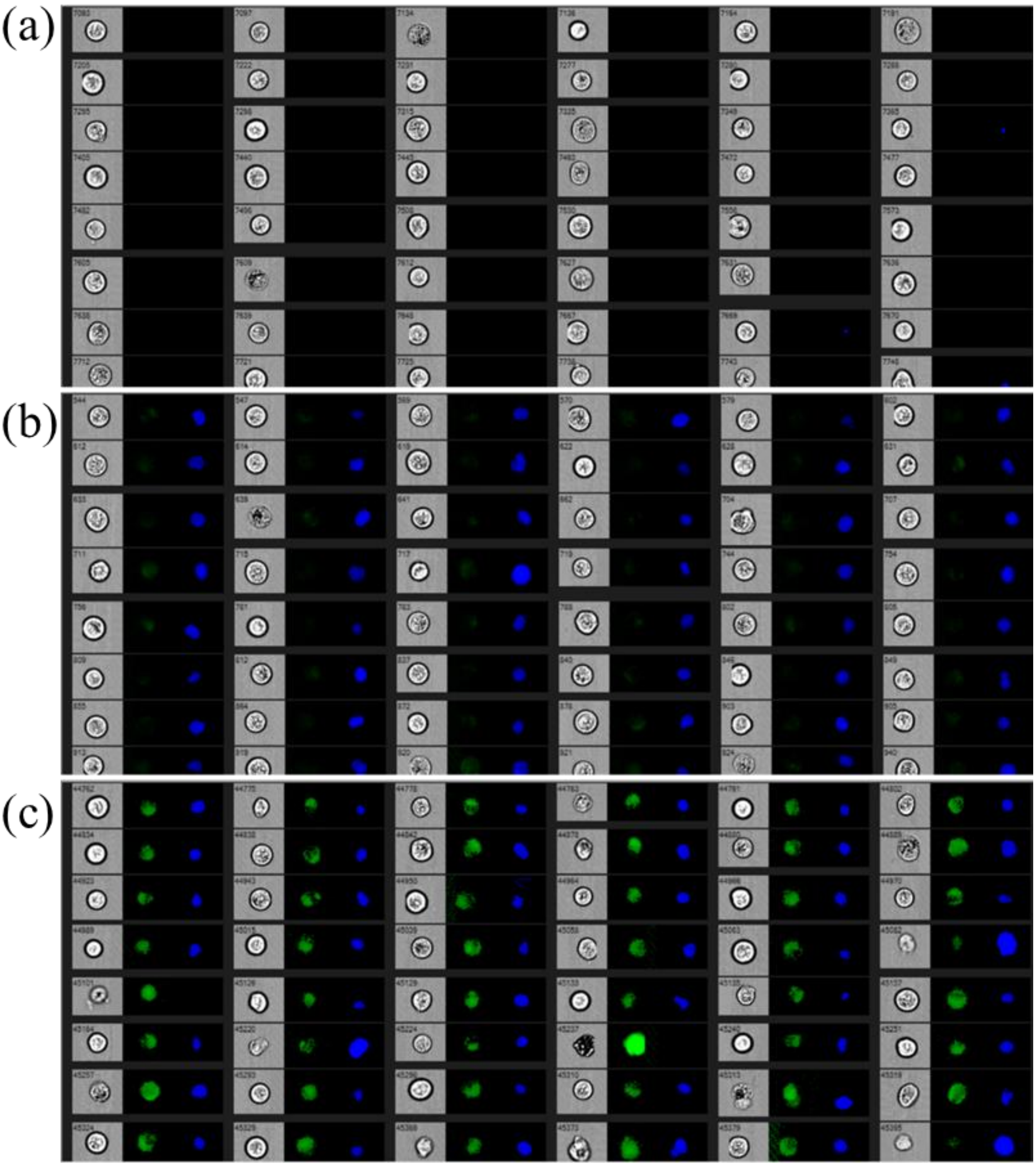

Fig. S10 Partial screenshot of imaging flow cytometry results: (**a**) untreated cells, (**b**) cells with stained nuclei, and (**c**) cells stained with nuclei and treated with the Anti-CEA-CAT-ZIF-8. The morphology of each cell was recorded with ultra-violet light (first column). The nanobots are labelled with FITC (green, second column) and the cell nuclei are stained with Hoechst (blue, third column)

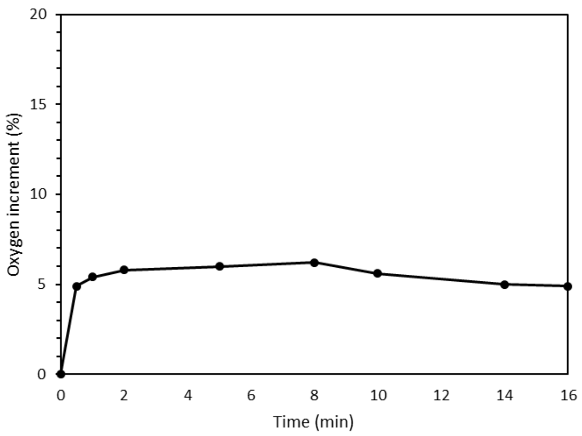

Fig. S11 Dissolved oxygen increments in the working environment

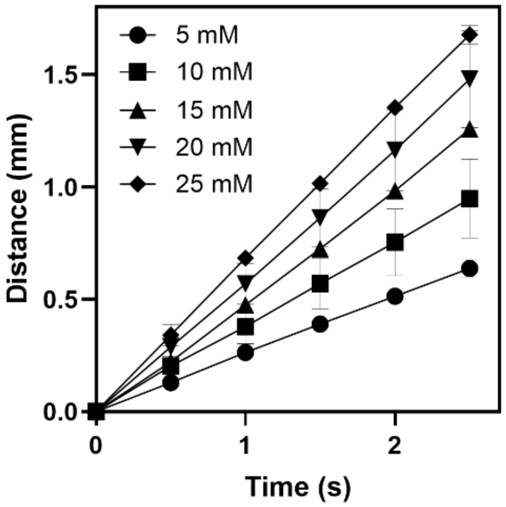

Fig. S12 The displacement of the Anti-CEA-CAT-ZIF-8 nanobots with different concentration of hydrogen peroxide. The error bars represent the standard deviation for three independently recorded trajectories

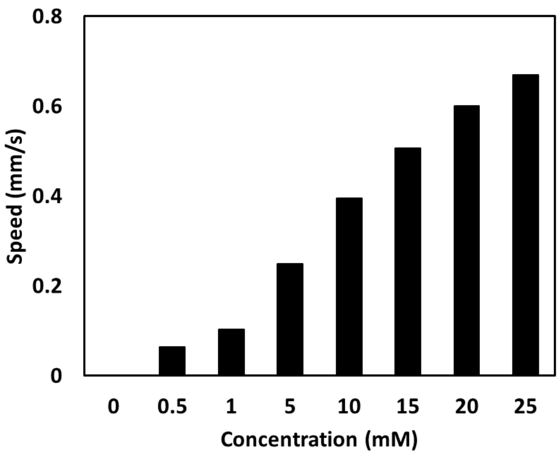

Fig. S13 The average ascending velocity of the Anti-CEA-CAT-ZIF-8 nanobots with different concentrations of hydrogen peroxide

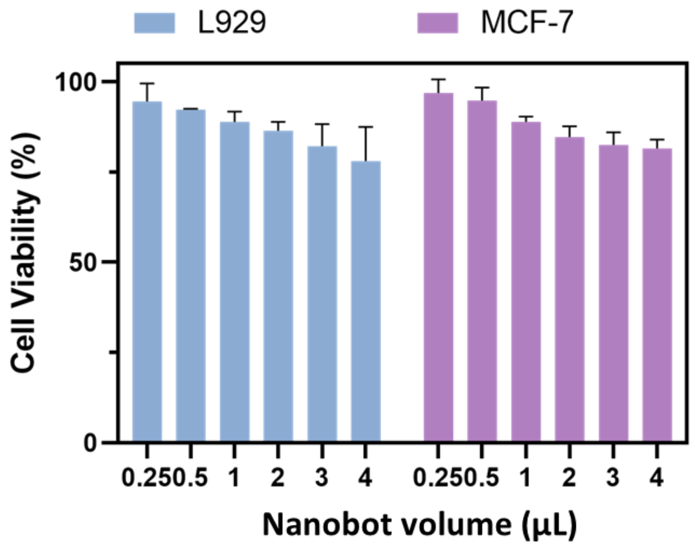

Fig. S14 Cell viability (CCK-8) with different amounts of Anti-CEA-CAT-ZIF-8 ranging from 0.2 to 4 μL

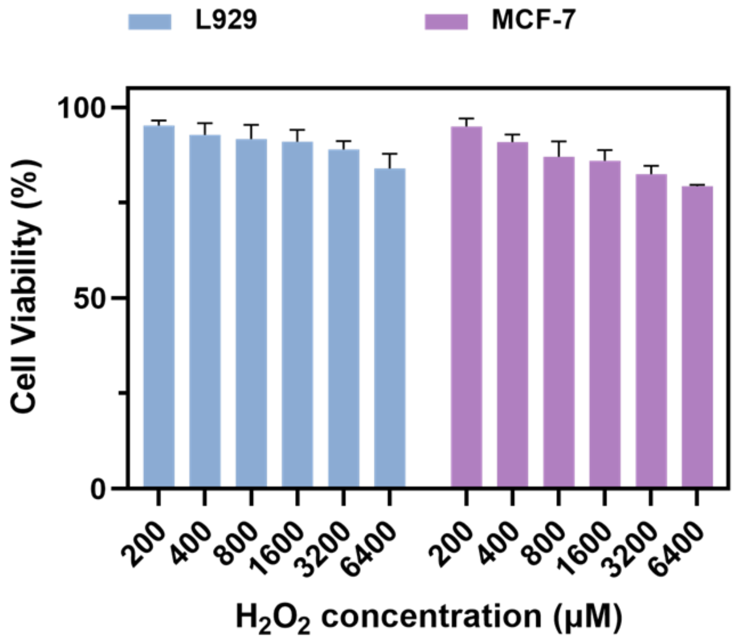

Fig. S15 Cell viability (CCK-8) with fixed amount of 0.5 μL of Anti-CEA-CAT-ZIF-8 and different amounts of H_2O_2 ranging from 0.2 to 6.4 mM

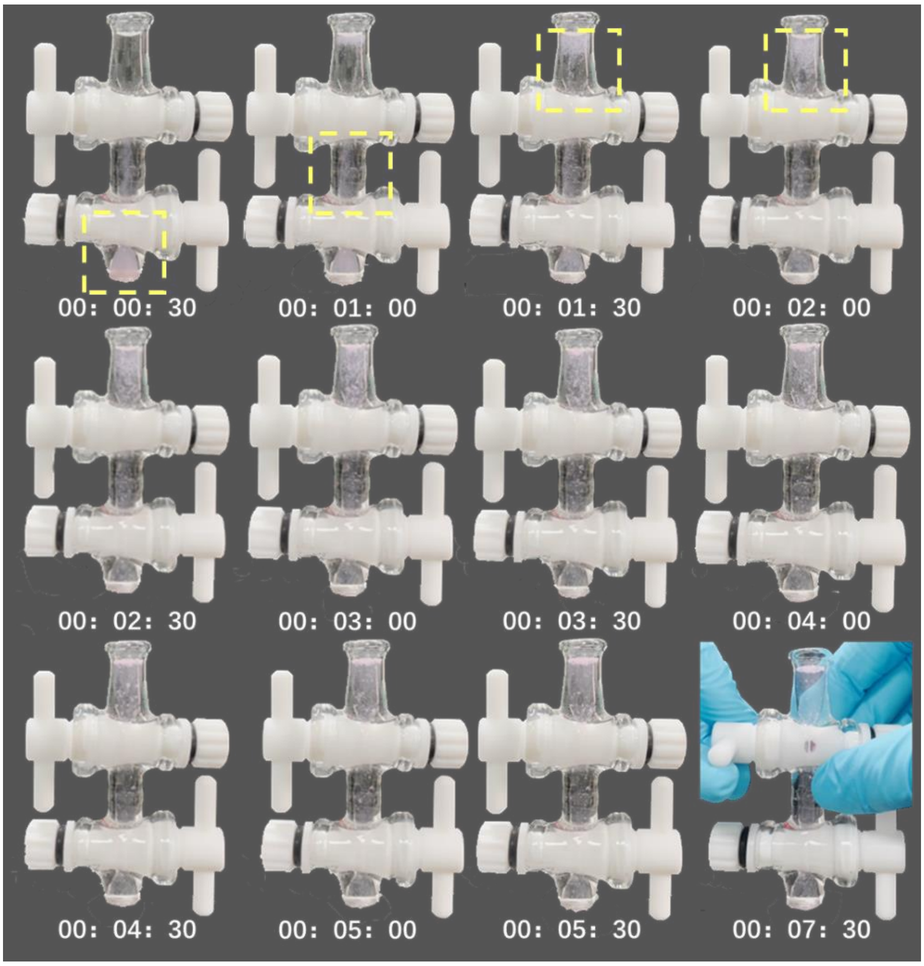

Fig. S16 Screenshots of the actual path of nanobot attached cells in the customized glass column extracted from the video. The positions of the nanobots are indicated with the yellow dashed line

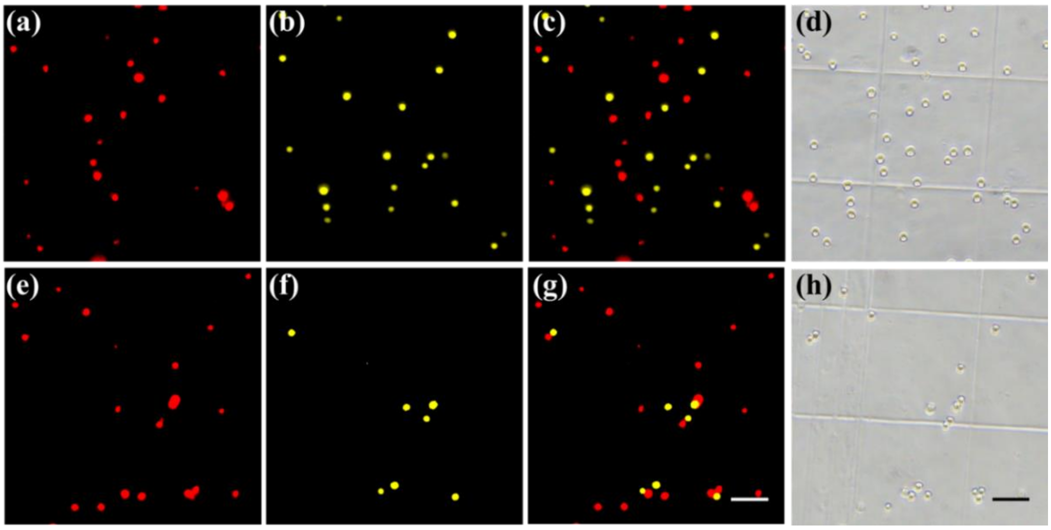

Fig. S17 Fluorescence images of (**a-d**) before and (**e-h**) after nanobot-enabled cell "find-and-fetch" from adherent cell mixture. The MCF-7 (pseudocolored in red) and L929 (pseudocolored in yellow) were dyed with Hoechst and calcein AM, respectively. Scale bars are 200 µm

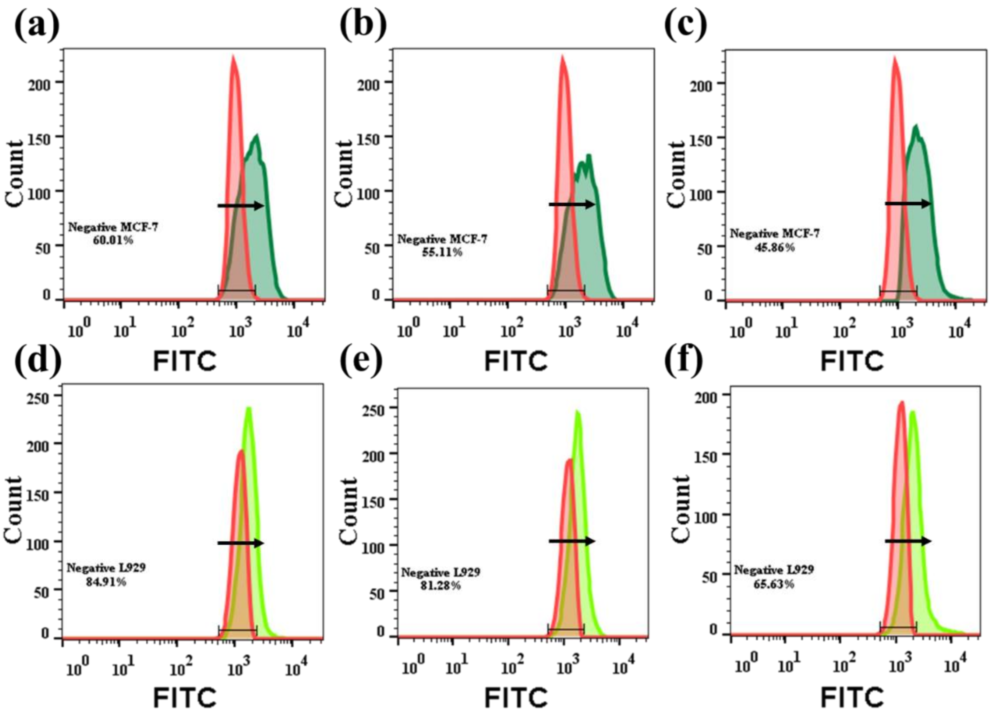

Fig. S18 Flow cytometry spectrum shift with (**a, d**) 1 μL, (**b, e**) 1.5 μL, and (**c, f**) 2 μL of FITC-labelled Anti-CEA-CAT-ZIF-8. The spectra of pure cells were displayed in red. The shifted spectra of MCF-7 and L929 are displayed in dark green and light green, respectively

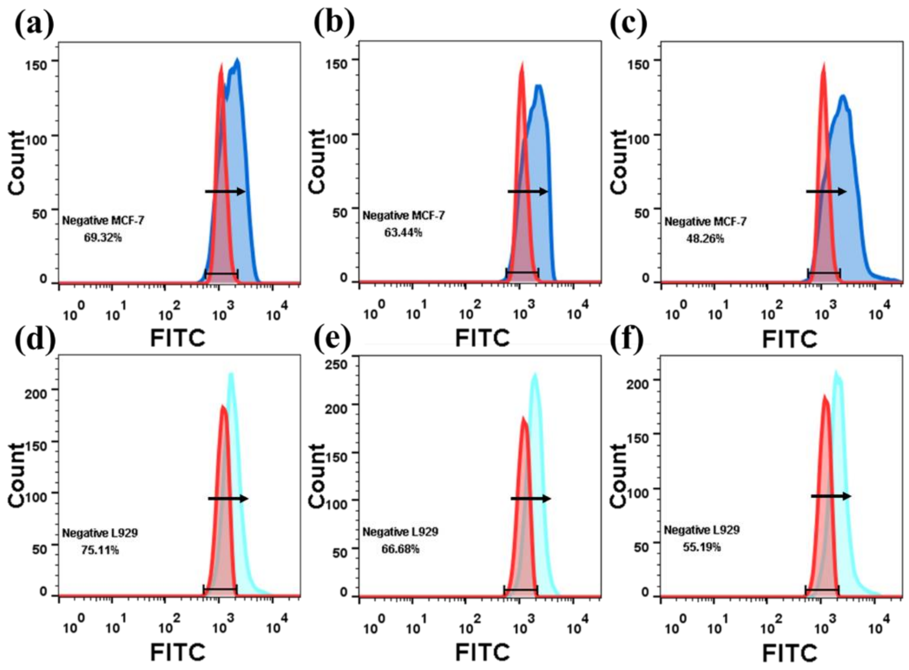

Fig. S19 Flow cytometry spectra shift with (**a, d**) 1 μL, (**b, e**) 1.5 μL, and (**c, f**) 2 μL of CAT-ZIF-8. The peaks of pure cells are displayed in red. The shifted peaks of MCF-7 and L929 are displayed in dark blue and light blue, respectively

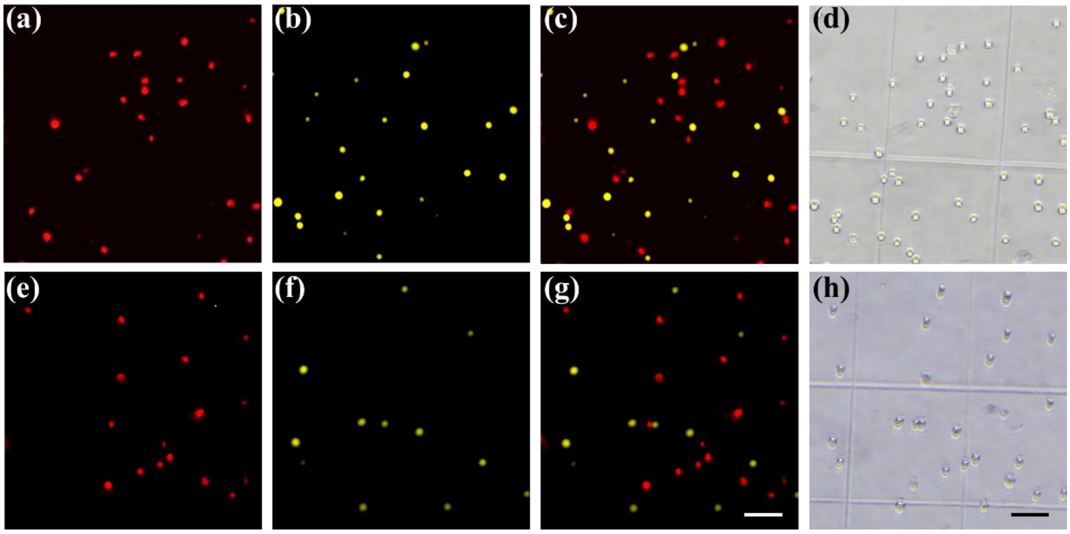

Fig. S20 Fluorescence images of (**a-d**) before and (**e-h**) after nanobot-enabled cell "find-and-fetch" from suspended cell mixture. The MCF-7 (pseudocolored in red) and L929 (pseudocolored in yellow) are dyed with Hoechst and calcein AM, respectively (Scale bars are 200 μm)

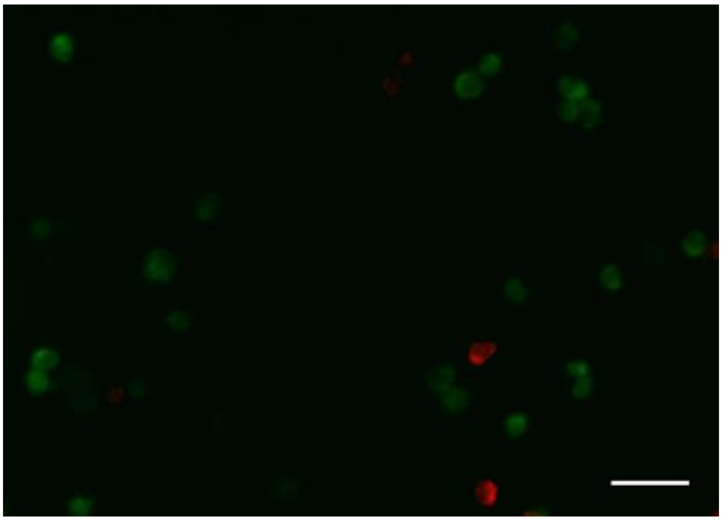

Fig. S21 Fluorescence image of lifted cells from mixed L929 and MCF-7 cells in the ratio of 1:1000. The L929 and MCF-7 are labelled calcein AM (green) and with Celltracker Deep Red (red), respectively (Scale bar is 50 μm)

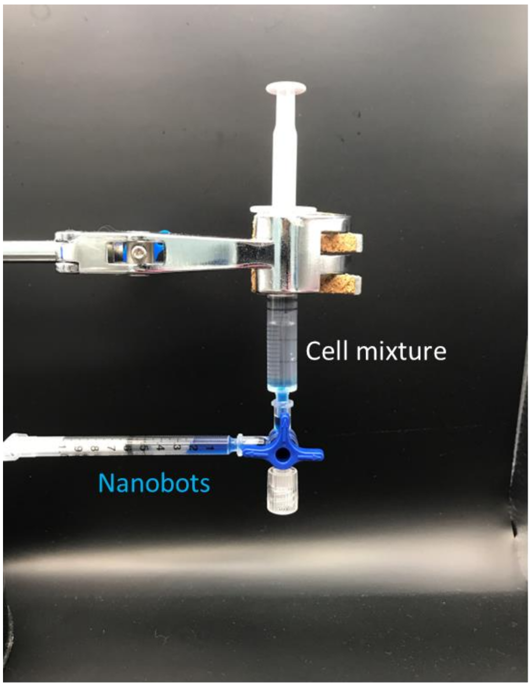

Fig. S22 A disposable syringe attached to a three-way valve for cell separation

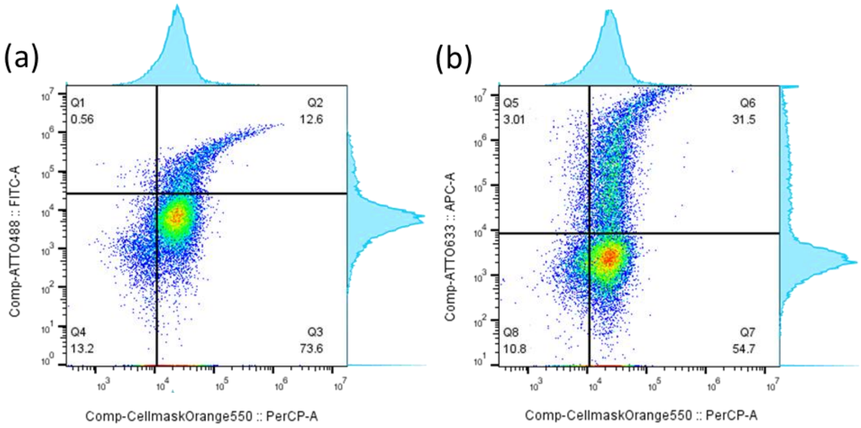

Fig. S23 Flow cytometry results of lifted cells from mixed L929 and MCF-7 cells in the ratio of 50%:50%. The MCF-7 cells are labelled with Cellmask Orange

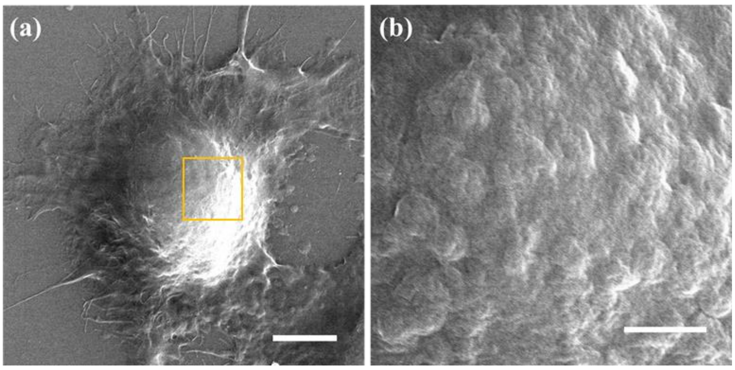

Fig. S24 SEM images of the recovered cells after re-seeding. Scale bars are 5 μm in (**a**) and 1 μm in (**b**)

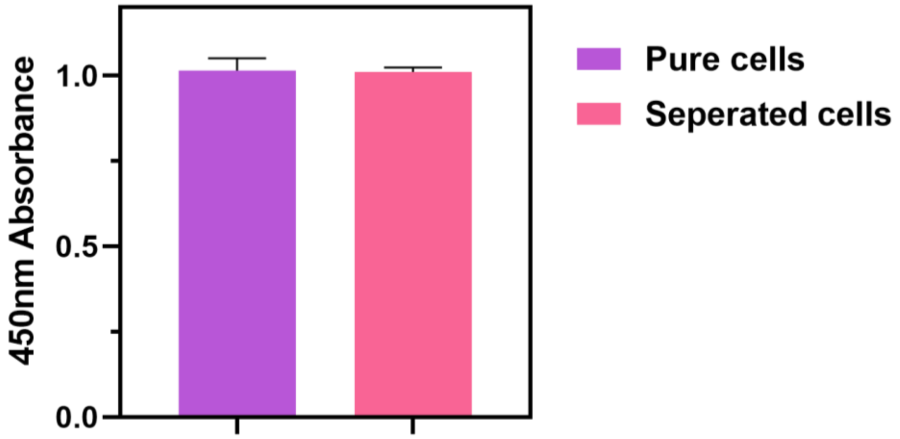

Fig. S25 Cell viability (CCK-8) of pure cells and recovered cells after nanobot-enabled "find-and-fetch"

Supplementary References

[S1] K. Liang, R. Ricco, C. M. Doherty, M. J. Styles, S. Bell, N. Kirby, S. Mudie, D. Haylock, A. J. Hill, C. J. Doonan, P. Falcaro, Biomimetic mineralization of metal-organic frameworks as protective coatings for biomacromolecules. Nat. Commun. **6**, 7240 (2015). https://doi.org/10.1038/ncomms8240

[S2] C. Yim, H. Lee, S. Lee, S. Jeon, One-step immobilization of antibodies on ZIF-8/Fe3O4 hybrid nanoparticles for the immunoassay of Staphylococcus aureus. RSC Adv. **7**, 1418 (2017). https://doi.org/10.1039/C6RA25527B

[S3] R. Alonso, P. Jimenez-Meneses, J. Garcia-Ruperez, M. J. Banuls, A. Maquieira, Thiol–ene click chemistry towards easy microarraying of half-antibodies. Chem. Commun. **54**, 6144 (2018). https://doi.org/10.1039/C8CC01369A
